# The Denied Pleasure of Eating: A Qualitative Study with Functionally Diverse People in Spain

**DOI:** 10.3390/foods10030628

**Published:** 2021-03-16

**Authors:** Carmen Cipriano-Crespo, Borja Rivero-Jiménez, David Conde-Caballero, F. Xavier Medina, Lorenzo Mariano-Juárez

**Affiliations:** 1 Faculty of Health Sciences, University of Castilla La Mancha, 45600 Talavera de la Reina, Spain; MariaCarmen.Cipriano@uclm.es; 2 Polytechnic School, University of Extremadura, 10003 Cáceres, Spain; brivero@unex.es; 3 Faculty of Nursing and Occupational Therapy, University of Extremadura, 10003 Cáceres, Spain; lorenmariano@unex.es; 4 Faculty of Health Sciences/Foodlab & Unesco Chair on Food, Culture and Development, Universitat Oberta de Catalunya（UOC), 08018 Barcelona, Spain; fxmedina@uoc.edu

**Keywords:** disability, pleasure, food, qualitative research

## Abstract

This qualitative study explores the difficulties in experiencing eating-derived pleasure within a group of functionally diverse people, based on personal interviews and Grounded Theory. Understanding the feelings and subjective experiences of functionally diverse people can help develop new approaches to address their loss of pleasure and motivation regarding food intake. The study included 27 participants, aged between 18 and 75 years, all of whom had a functional deficiency that affected the occupational aspects of the eating process. Interviews were conducted in clinical settings and several centres for differently abled people. Four main themes emerged from the analysis: eating through obligation; fear of eating; the social life of food; and the importance of the taste and visual aesthetics of food. These themes underscore the importance of taking into account the phenomenological experiences of pleasure in the eating process.

## 1. Introduction

Spinoza defined desire as “the very essence of man”. However, long before his time, Epicurus had argued that desires should not be indulged without limitation, but wisely and soberly. While he considered eating and drinking as natural and necessary desires, yearning for extravagant foods without moderation was not. The concept of nutrition that developed within nutritional science during the 20th century was focused on calories and macronutrients, reducing what is a cultural construct to its purely biological aspects. Following on from this viewpoint, approaches to food within health sciences became Epicurean: while nourishing the body was a natural need, the pursuit of certain desires and pleasure was unnecessary.

By contrast, the perception of nutrition from an anthropological perspective is based on social relationships. Therefore, its interest does not lie in issues such as nutrients or boosting the metabolism [1]. It focuses, instead, on food consumption, commensality, and other cultural categories that help define a person as a social being. As Contreras [2] argues, “food is more than a collection of nutrients chosen according to a purely dietetic or biologic rationale [...] Eating is a social and cultural issue, while nutrition is a physiological and health one” (p. 11). However, until very recently, biomedical approaches to food have been based on a biological determinism that marginalised sociocultural aspects and experiences, disregarding them as epiphenomenal [3,4] and as accessories within the epistemological discourse [5]. Accordingly, the analysis of eating habits was focused on body nutrients—an approach that validated nutritional science’s quantitative methodologies as more scientific.

This reductionistic approach to body health, however, ignores the central role played by food in many human activities [6]. Indeed, health professionals’ approaches to food have, until very recently, focused almost exclusively on nutritional issues—in terms of caloric intake and macronutrients. Massana [7] defined this professional approach as “medicalising” food, making patients eat “nutrients” instead of “food”. This focus on the nutritional properties of food often resulted in the neglect of smells, flavours, personal tastes, and eating-derived pleasure. However, while eating is undoubtedly an essential activity for human survival, anthropology has shown the importance of cultural mandates in defining what is eaten, by whom, and in which contexts [8]. All these practices define particular and specific ways of existing within the world and are essential components of social life.

As Simone Weil argued, the loss of pleasure leaves people devoid of energy and resources, which makes it difficult to perceive the positive aspects of life [9]. Food preferences—the types and amounts of foods people consume—are influenced by the pleasure that we expect them to yield [10]. Experiencing eating-derived pleasure depends on the different, interrelated dimensions of pleasure—sensorial, interpersonal, and psychosocial [11]—the combination of which makes this experience a fundamental aspect of food choices. The perception of food-related pleasure involves three aspects, each one of them associated with a distinct underlying neurobiological mechanism [12]: the elements of motivation (wanting a certain food); liking (a neural reaction to the hedonic impact of taste, which causes pleasure); and learning (predictive associations based on positive past experiences of food) [13]. Huergo [14] noted that “the food and beverages a person consumes leave traces in them, be they organic—i.e., nutrition—or sensory—i.e., pleasure and other feelings” (p. 1148).

It has also been argued that human dietary patterns can be affected by emotions—people’s feelings have an impact on their food preferences, shaping their choices as well as the amount of food consumed [15]. The analysis conducted by Lyman [16] suggested that people experiencing positive emotions tended to eat healthier foods, while the consumption of junk food was greater among people experiencing negative emotional states. Moreover, food has the capacity to elicit past memories and emotions—to evoke flavours and pleasures experienced in the past, as well as feelings towards those with whom those meals were shared. As Hernández [17] noted, “a plate of food can bring back to the present people, times, and places; evoke images and sensations; condense emotions and feelings, either good or bad; and bring together or drive away cooks and guests” (p. 244). So-called “hedonic hunger” is thus a crucial aspect of food intake: human appetite is driven by pleasure and palatability, and not just homeostatic eating motivations [18].

Food-derived pleasure, however, has often been neglected or marginalised in favour of approaches that prioritise health issues—as if enjoyment and pleasure were not compatible with health [19]. On the contrary, it has been suggested that deriving pleasure from the food consumed is a key aspect in fostering healthy eating habits [20]. The choice of food and the social and physical environments in which it is consumed all have an effect on hedonic eating experiences. However, these experiences are also influenced by internal conditions, such as individually specific characteristics and motivations [21]. Eating is an important source of pleasure (hedonism), which might contribute to happiness [21]. For some people, a delicious meal consumed in the right social context can provide enormous pleasure [6].

As the anthropologist Marta Allué suggested in her autoethnography [22], when the sole purpose of food intake is acquiring the nutrients necessary for the body, mealtimes can soon turn into a torment. Allué described the experience of being fed through a nasogastric tube while she was hospitalised in a burn unit, which was far from pleasurable—rather, it created a hateful sensation, as the food accumulated in her stomach without her being able to taste it.

As Cavalieri [23] has noted, humans are the only beings who have transformed a physiological need into a pleasurable cultural experience, laden with meaning. Food can be a great source of pleasure, not only through its consumption, but also during its acquisition and preparation [24]. However, certain difficulties caused by health conditions might hinder the experience of food-derived pleasure.

The main aim of this study is to explore issues associated with a loss of pleasure and motivation in relation to food within a group of differently abled people.

## 2. Materials and Methods

### 2.1. Procedure

This qualitative study is based on Grounded Theory, a research method aiming to develop theory that is “grounded” in systematically collected and analysed data, in order to explain social processes in their specific contexts [25]. An inductive approach was followed for data collection and analysis [26], aiming to understand and create categories from raw data through content analysis. The constant comparison method was used for identifying similarities and differences and creating categories from the empirical data collected. This allowed for a better analytical understanding of the participants’ experiences (illness)—functionally diverse individuals that presented with feeding difficulties.

In this study, the use of qualitative description enabled the authors to remain close to the data, minimising research bias and developing a rich, straightforward, and accurate understanding of the topic based on the participants’ own words, expressed in their own settings [27,28], bearing in mind, at the same time, that the different sets of methods used in qualitative research have their own characteristics, perspectives, and applications [29,30,31,32].

### 2.2. Participants

Our study included 27 participants (21 males and 6 females) aged between 18 and 75 years, all of whom had a condition (acquired brain damage, amyotrophic lateral sclerosis, cancer, Duchenne muscular dystrophy, multiple sclerosis, Niemann–Pick disease, spinal cord injury, or stroke) that caused functional deficiency in the occupational aspects of the eating process (Table 1). Some of them presented with problems related to chewing or swallowing food, some were unable to use cutlery or feed themselves and needed help, and some were tube fed.

All subjects participated voluntarily in the study, signing an informed consent form once the study’s main goals were explained. Individuals with cognitive impairments due to dementia and/or intellectual functional diversity, those who did not provide written consent, and those who refused to be recorded were excluded from the study.

### 2.3. Data Collection

The data collection methods included in-depth interviews based on semi-structured, open-ended questions (Table 2). This flexible approach allowed us to make adjustments and explore new themes that emerged over the course of the interviews. Interviews were carried out between January and December 2019 in different settings (speech therapy clinics, hospital rehabilitation units, and organisations for functionally diverse people). All were audio recorded, with field notes taken for their duration. Particular care was taken to ensure the participants’ comfort and privacy during the interviews, to guarantee an honest, openhearted conversation between participants and researchers. All interviews were conducted by a single researcher, lasting between 60 and 120 min.

In order to preserve the participants’ privacy and confidentiality, all personal data that could reveal their identity have been eliminated from this article. The original audio recordings were assigned individual code names before the analysis started, and were destroyed once they had been transcribed.

### 2.4. Data Analysis

All interviews were fully transcribed, and their content analysed in tabular datasheets. The empirical data collected were analysed and interpreted in light of current social theory and theoretical occupational models. Content analysis was conducted by two researchers, working independently. Both had extensive experience in qualitative research. Once their analysis was completed, their results were shared with the rest of the research team, who assessed their adequacy. The participants’ interviews were analysed through systematic content-coding: first, preliminary units of analysis were established after an initial reading of the transcripts; then, using the constant comparison method, data were grouped into concepts, generating categories accordingly. A total of 22 categories were initially defined, which were finally reduced into 10 categories. Once the data had been broken down into categories, relationships between categories and subcategories were actively and systematically analysed in a process of axial coding [33]. Finally, a process of selective coding was followed, with categories being grouped into main themes. These themes capture and describe the participants’ most salient experiences and how they are perceived.

To guarantee data quality, validity, and accuracy, several triangulation techniques were applied: (1) across researchers—with interviews being conducted and analysed by different specialists; (2) across methodologies—with the participants’ data gathered using a variety of techniques (in-depth interviews and field notes); and (3) across the different theoretical approaches on which the inductive method was based.

To guarantee the quality and validity of the study, throughout their research, the authors worked in line with the Consolidated Criteria for Reporting Qualitative Research COREQ criteria and verification checklist for reporting qualitative research [34].

### 2.5. Ethical Considerations

This study was conducted in accordance with the ethical principles outlined in the Declaration of Helsinki and the Belmont Report. This research protocol was approved by the Clinical Research Ethical Committee of the Talavera de la Reina Integrated Management Area (CEIm del AGI de Talavera de la Reina in Spain, Nuestra Señora del Prado Hospital, ref: 18/2014).

## 3. Results

**Figure 1:**
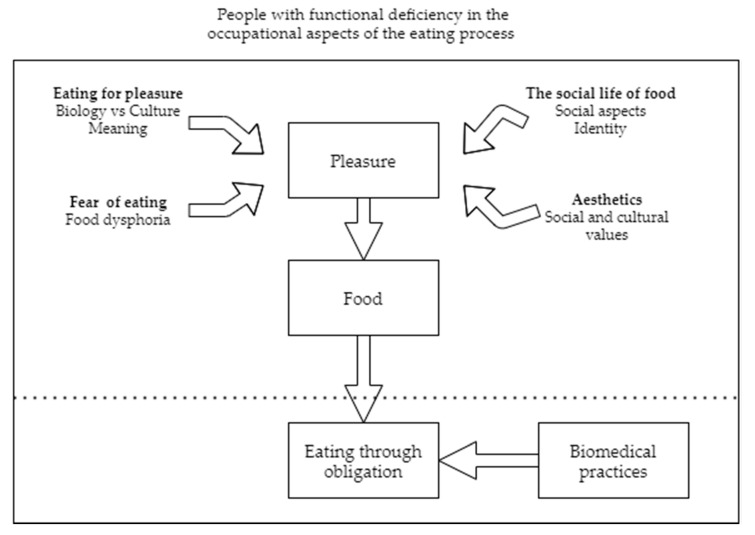
Key Themes.

The analysis of the empirical data suggested four key themes (Figure 1), which underscored the crucial importance of pleasure in the eating process, and how the loss of this pleasure for people with functional diversity can trigger changes in the context and the way in which food is consumed.

### 3.1. Eating for Pleasure vs. Eating through Obligation: Biology vs. Culture

In the current Western social and cultural context, the enjoyment of food is usually one of the factors related to food intake. However, eating can sometimes be an imposition and, when the only reason for eating is obligation, it might give rise to significantly negative emotions and feelings. This creates a gap in perception between, on the one hand, those who can choose what they want to eat and for whom—with a gourmet approach—food is a means to experience different sensations; and, on the other, those for whom “the key issue is to fill their stomachs through artificial feeding—eating only what is possible” ([35], p. 2). This experience was noted by six out of the 27 participants in our research who were being artificially fed. Their present situation made them feel different to how they were before the onset of their condition and reduced their capacity to enjoy food and eating. One such example is Leoncio, who underwent a laryngectomy as a result of laryngeal cancer. He could not enjoy food because he was being fed through a syringe connected to a feeding tube—he could neither taste nor smell the food: “[...] I thought it would get better eventually, but the pain I was experiencing made eating a struggle—whereas, for me, food had always been a source of pleasure”. While his stomach might have been full, his mind, body, and soul did not feel nourished.

In clinical contexts, healthcare professionals regularly pressurise patients into eating to meet the nutritional needs necessary for survival, with this aspect often becoming the only issue under consideration [36]. For these patients, however, having to eat without enjoying the process—sometimes without wanting to eat at all—might increase the suffering caused by their new life situation. Antonio, another of the 10 participants who underwent a laryngectomy due to laryngeal cancer, stated that eating through obligation was the worst thing that could happen to a person:

“It was really awful, I knew that I had to start eating and then they would take the tube off, but it was very painful and I did not want to—they were all pushing me to do it, I felt like a child, and in the end it was my mum who always had to feed me–otherwise I would hardly eat at all”.

Antonio did not just want food to make him feel full—he also wanted to be able to savour the food he was consuming, to enjoy what is one of the great pleasures in life. Indeed, as Kringelbach [12] suggests, although food is clearly essential to survival, what makes eating worthwhile is the pleasure that it elicits. However, for people suffering from dysphagia, for whom eating is a nutritional obligation, and for those who are tube fed and unable to enjoy the smell and taste of the food, or social company at mealtimes, all sense of pleasure is lost. As such, the food of the past, of a time when meals could be created and shared in contexts laden with emotional meaning, is transformed into an imposition.

A total of 20 out of 27 participants noted that their current meals did not take into consideration their tastes and preferences. Instead, they were based on which foods were easier and safer to eat. As a result, it was difficult to experience any food-derived pleasure, with some admitting that this pleasure had been completely lost. Moreover, eating food that they did not like as a result of external pressure made it more difficult to digest. The changes caused by their condition did not only affect their bodies, but also had an impact on their culinary normality. Their cravings for food went beyond the anticipation of a pleasurable experience, to the point of sometimes being painful physical sensations. As their bodies now needed help, the eating process had lost its enjoyable aspects, and created disaffection—it embodied the pain caused by the culinary restrictions and the awareness that they only ate because they needed to. This was the case for Paloma, who had amyotrophic lateral sclerosis, and was unable to consume many of the foods that she used to enjoy. She was living in a care home for the elderly, where she was given what she called “shitty soups”—soups which were prepared especially for her.

“They all taste and smell the same, with a thick, paste-like texture that instinctively makes me want to reject it; however, the hunger is stronger. I feel pity for myself, I am disgusted by the food I am given, but I still eat it”.

It could be said that Paloma ate purely because of a biological need, and at no point was her need to derive pleasure from her food fulfilled. This made her feel discontented with herself, as the need to satisfy her hunger with no regard for the taste of her food felt almost animalistic. Every time she had to give in and eat something her senses rejected as unappetising, she felt a pain—as if she was relinquishing the person she was before her illness.

### 3.2. Fear of Eating. Notes on Food Dysphoria

Difficulties in the eating process that appear as a consequence of new life situations require functionally diverse people to devise alternative approaches to food—to introduce adjustments that can be either temporary or permanent. These adjustments affect the types of food that can be eaten, how they are prepared, and how they can be consumed.

For Tomás and seven other participants who underwent a laryngectomy during their treatment for laryngeal cancer, problems swallowing food persisted after their discharge from hospital. Tomás had to be extremely careful because he often choked on his food, and to avoid this he had to eat very small mouthfuls and chew for a long time:

“I feel really scared every time I eat, in case I choke and just die—even though at first I would not have minded if I had died, when you are so close to it you really cling to anything”.

For people experiencing a fear of eating, each meal can be perceived as a challenge, a form of torture, which creates suffering and transforms the eating process into a mechanical act. Shifting this perception so that patients can derive enjoyment and pleasure from their food again is an arduous process that requires considerable effort and care. At the same time, food experiences for functionally diverse people might still feel incomplete, as some foods might be banned or considered dangerous.

Mealtimes, which might have been routine before, were now challenging and stressful processes, required more energy and effort, and were weighed down by concerns about—or even fear of—dysphagia [37], as noted by 22 out of our 27 participants. That was the case for Paloma, who had amyotrophic lateral sclerosis. She had to give up on many of her favourite foods, such as rice with chicken, pizza, or lasagne, among others, due to her fear of choking. As she had to sacrifice pleasure for the sake of safety, her eating experiences felt incomplete—most foods were deemed too dangerous for her to eat.

The participants in our study described the apprehension they felt after experiencing dysphagia, and how it had limited their choices. They had become unable to eat or drink what they would like to—only what they could or were allowed to. Arturo, who was tetraplegic, noted:

I found it very hard to eat and swallow while I had a tube on, it was unpleasant and I was afraid of choking.

Clinical interventions aimed at reducing the fear of choking when swallowing, although intended to help, can instead cause or increase rejection of food. Fernando, a participant with laryngeal cancer, was prescribed a fluid thickener to reduce his risk of choking. This, however, caused a conflict between his thirst and the revulsion he experienced when drinking—with the latter eventually prevailing, as an expression of the power of culture (taste) over physiology: “I understand that [not drinking] was not good for me, but the disgust I felt for the thickener was too much for me”.

Ultimately, the emotional weight of the loss of eating-derived pleasure, together with the distress experienced while eating, might have an impact on the social aspects of food intake, thereby reducing the opportunities for sharing meals with others, and thus the social pleasure gained from partaking in food consumption and conversation. This increases the sense of loss even further.

### 3.3. The Social Life of Food

Some of our participants recalled the difficulties they experienced in enjoying their meals because of the environment in which they took place—spaces devoid of emotion, which, in turn, affected the perception of taste, and the general enjoyment derived from food. Often, these meals took place in dining rooms lacking aesthetic components that could enhance their sensory experiences of food. The excessive background noise (i.e., televisions); the white, impersonal walls; sad and ugly tables that are too narrowly spread out, thus turning the eating process into a group activity, where mastication or swallowing difficulties were magnified because of public exposure and lack of privacy. Paloma, because of problems caused by her condition (amyotrophic lateral sclerosis), was living in a care home for the elderly. As she experienced severe dysphagia, she did not eat with the other residents. She took her meals in a different room, alone, except for the assistant who helped her—as she could not manage on her own.

“They give me my meals on my own, in a sad room with only four tables randomly distributed. It is not a nice room—it is cold, and I do not want to spend much time there–sometimes I do not even feel like eating at all, because I do not feel comfortable there. It is at times like that when I miss my dining room at home even more, and the sadness and longing I feel make the care home’s dining room look even worse”.

A total of five of our participants were interviewed in the hospitals in which they were being treated—spaces characterised by a high degree of internal organisation [38], where patients have very limited decision-making capacity regarding food. In one of the hospitals where our study was conducted, there was only one dining area, located on the top floor and accessed by a ramp. This dining area was only for patients who did not require help to eat, as there was only one support assistant on duty at a time (to help if cutlery was dropped, or water jugs needed to be refilled, etc.). As this support assistant was unable to help those who could not feed themselves, impaired patients were given their meals in the isolation of their own rooms. Hospital patients lucky enough to receive visits from relatives would get help from them with their food (Hospital patients who were accompanied by a relative 24 h a day were privileged in that they were not alone in their rooms all day, as other patients were. For these other patients their only help and company came from support staff, who never had as much time for them as the patients would have wished.), but the rest had to wait to be fed by support staff.

Although dependent patients did not eat in the common dining room, their hospital rooms did not have a dedicated space for eating. Therefore, they had to use a worktop placed along the wall, which served a variety of uses, e.g., as a place to leave patients’ clothes or laptops, but also where staff left pillows and bed linen while they helped the patient get changed and tidied up the room, sometimes causing hygiene issues. In this context, mealtimes were seen as just another task within the patients’ rehabilitation schedule, which undoubtedly did not contribute to them being perceived as pleasurable.

Dependent patients were thus confined to an enclosed space, practically facing a wall. Moreover, although they sometimes ate at the same time as other patients they shared their room with, their seating arrangements did not facilitate social interaction: the patients did not sit across a table from each other but along the same worktop, with the support assistant who helped with their food standing next to them, making interaction difficult. This was very different from the culinary normality experienced in a canteen or dining room, and it contributed to creating the sense that their conditions excluded them from their peers.

However, those participants who went to the hospital’s dining area for their meals did not fare much better in terms of enjoyment, as this was not a warm room that invited an appreciation of food. It was a large room, with several tables placed close to each other, large windows along one wall that allowed a glimpse of the outside, and a solid wall on the other side, which had been half-heartedly decorated with pictures that were proportionally too small, thus contributing to the sense that the room was too large to be welcoming. It was a soulless room, which some of our participants even described as shabby or cheap. It had no television and hardly any decoration, nothing to inspire patients to talk about their experiences of food. As one participant, Dioni, said, it was a space only fit “for eating–sadly”, a space that did not provide sensory or emotional nourishment, which increased the depersonalisation felt by patients. This absence of sensory values extended to the food consumed there, greatly reducing the scope for pleasure.

### 3.4. The Importance of Visual Aesthetics of Food

The palatability of food increases with its aesthetic appeal, as the visual impact of food presentation is a key factor in the pleasure derived from eating.

The aesthetic experience of food is also affected by the accompanying paraphernalia—cutlery, table linen, glassware, etc.—which affect the way in which food is perceived, and increase its visual appeal [5]. Tables covered with clean, hand-embroidered tablecloths; carefully curated tableware to impress guests; glasses that allow the liquids they contain to be fully appreciated; and a well-decorated and well-provided table (in terms of the amount and quality of the foods served) are crucial when food is to be offered to others. The containers in which food is served are also a key aspect in the perception of their taste. The study conducted by Piqueras-Fiszman et al. [39] concluded that even the colour of the serving dishes affected the way in which food was perceived. However, while consideration of the aesthetics of food might affect its acceptance, the taste of the food itself is crucial. For some of the participants in our study, this was a difficult goal, as the only food they could ingest, both in the selection of ingredients and the way in which the food was prepared, had sacrificed taste and aesthetics for nutritional content.

Solids often had to be substituted by puréed food, with unappetising textures and unappealing aesthetics. Even the colour of the food was reduced to a repetitive palette, further enhancing the loss of visual stimuli. This puréed food looked uninviting, and therefore patients had to make an extra effort to overcome its rejection. Although they understood that they needed to eat to continue their recovery, there was very little appeal in the food they were served.

Some of our participants were tube fed, and their situation, again, was different. They had no choice regarding the food and drink they were able to consume, and while fresh fruit, fish, meat, or bread might have been their preferred choices, they were aware that they would perhaps never taste them again. For them, the natural variability in the colour, shapes, sizes, and other features of food was completely lost, as their food came out of a plastic tub. Indeed, this kind of food is marketed as a formula, underscoring its nutritional, medical purpose—rather than aiming to be enjoyable or visually appealing. Another element to be taken into consideration is the uncertainty and apprehension caused by the process of tube feeding. For relatives trying to help these patients, the fear of causing them pain is an added stress factor, together with their lack of familiarity with the surrounding medical equipment and the new food intake process.

As our participants noted, cooked meals had been reduced to their most basic aspects. The memories of kitchen worktops covered in foods with different smells, colours, and shapes that could be savoured and enjoyed had become a thing of the past. The noises of cooking, cutlery and pans had been replaced by food that came out of cans or plastic containers, to be consumed straight away or prepared in only a few minutes, and whose main goal was optimising nutritional intake. Gone too were tablecloths and linen napkins. Their current meals were unattractive and unappetising, with a focus on functionality and not on providing a pleasurable experience.

The new visual aesthetics at mealtimes also included rubber tubes, droppers, and containers. Their food was acquired in pharmacies rather than supermarkets, and was not meant to be savoured. Being fed straight through a tube affected not only the aesthetics, but also the emotional perception of the room where the food was consumed. These were medicalised environments, where the aesthetics and pleasure derived from food had disappeared altogether.

## 4. Discussion

The analysis of our participants’ accounts highlights the importance of eating-derived pleasure for functionally diverse people, and how the physical difficulties they experience affect not only their eating patterns, but also their perception of food. Many of their narratives coincide with stressing how the difficulties caused by the progress of neurodegenerative disorders, spinal cord injuries, laryngeal cancer, or other rare conditions had turned eating into a challenge that could not be enjoyed—perceived instead as an obligation they had to go through to fulfil certain nutritional goals, without any consideration for pleasure. However, as Contreras (2) has argued, people eat for their own satisfaction, not that of nutrition experts. Previous studies have emphasised the relevance of food and eating-derived pleasure. Johnson et al. [40] suggested that the loss of this pleasure could be one of the criteria to consider as a predictor of the readiness to accept a gastrostomy among patients diagnosed with amyotrophic lateral sclerosis.

A large percentage of our participants indicated that their loss of hedonic pleasure had limited their food intake and their participation in social gatherings and celebrations involving food. Some had reorganised their daily routines to accommodate their new feeding needs. The study conducted by Block et al. [41] stressed the relationship between food, society, and culture, suggesting that pleasurable eating experiences were closely associated with positive relationships with food—psychological, physical, emotional, and social [42]. This study pointed out that a reduction in or loss of eating-derived pleasure had a significant impact on people’s physical and mental well-being, and also affected their social relationships—an issue also noted among the participants in our study.

For tube-fed participants, who could not derive any sensory pleasure from food intake, their lack of a decision-making capacity with regard to food choices came to symbolise a loss of control over their bodies and their lives, as well as their independence. In their study of patients fed through a nasogastric tube, Barbosa and Freitas [42] noted, as we have in our study, that mealtimes had turned into unwanted impositions, and were a stress factor for these patients. Difficulties in the eating process, therefore, might transform the experience and significance of food intake—an activity that loses its pleasurable connotations as it turns into a challenge that requires constant effort and care, while embodying the patients’ loss of control over their lives [43,44]. The changes associated with their new life situation also give rise to feelings of anxiety and fear in relation to food intake, which are also contributing factors in reducing food-derived pleasure [37]. 

The physical contexts in which food consumption takes place also have a significant impact on whether food is accepted or rejected, and whether a meal is started and fully completed. Meiselman, Hirsch, and Popper [45] argued that situational aspects were key to food acceptance and consumption: even when the food provided was exactly the same, the location in which it was consumed could alter the sensory pleasure it yielded. Indeed, there is a sensory aspect to eating-derived pleasure, which is enhanced when food is consumed in a warm and welcoming atmosphere [35]. However, the spaces where the participants in our study consumed their meals were and looked like hospital settings, rather than places intended to stimulate eating-derived pleasure.

Likewise, when food is presented in visually pleasing ways, in carefully considered serving dishes, the harmony created enhances the eating experience [46,47]. The attention to food aesthetics was a defining aspect of avant-garde cuisine, which sought to provide a multi-sensory, aesthetic experience in which the senses of taste, sight, smell, and touch combined to produce a sense of happiness through food [47]. However, the new eating situation of our participants involved a loss of culinary identity, materialised in the loss of “the products, cooking techniques, dishes, and consumption patterns that members of a cultural group consider their own, and others perceive as typical [of that group]” ([43], p. 1)—a culinary culture in which they could no longer participate. It is almost like a reversal of the civilising process, which made them feel different, and alienated from their former culinary practices, experiences, and narratives [48].

The disruption caused by their illness forced them to introduce changes to their eating habits, either temporarily or on a long-term basis. These adjustments affected what food was consumed, as well as how it was prepared, chewed, or swallowed and, being an involuntary imposition, this disruption transformed mealtimes into another of their daily chores.

For many of our participants, their new reality was also characterised by the use of new feeding tools, including syringes and feeding pumps. As well as having an effect on their bodies, these tools also symbolised and made them even more aware of the changes that had taken place in their lives. Barbosa et al. [42] have also noted how these new feeding techniques eventually contribute to changes in the patients’ social representations.

Not being able to eat what they would like to, how they would like, or with whoever they would like, caused longing and suffering for each meal that was missed, each mouthful that could not be savoured. It also caused longing for the days when eating was a pleasurable activity that allowed them to feel connectedness with friends and family through celebrations. Their present state not only limited their food choices, but also who their meals were shared with, a further imposition that hindered the (re)activation of the lost pleasure of eating.

## 5. Conclusions

The patients’ narratives examined in this study underline the importance of considering the phenomenological experiences of eating, while also focusing on nutritional intake. As our participants have noted, it is important to understand the value and significance of the disruption caused by an illness, and its physical and psychological consequences on the experiences associated with eating-related pleasure. Our participants’ accounts described how the range of foods they could or were allowed to eat was becoming increasingly restricted. The desire to experience pleasure from food sometimes led them to eat banned foods, despite knowing that, by doing so, they could be putting their lives at risk. Others sought alternative strategies to somehow recreate feelings of pleasure experienced in the past. Feelings of sadness and sorrow arose at having lost such an important source of pleasure, and the need to adapt their eating habits to their new life situation. The sadness at not being able to eat the food that they longed for was compounded by shame at not even being able to sit at a table with other people—shame at having to eat or be fed in a way that separated them even more from what they perceived as culinary normality.

The new eating strategies increased their isolation and loneliness, and the unappetising food and dissatisfaction they felt at mealtimes contributed to reducing their appetite further. In some cases, it disappeared altogether.

The loss of eating-derived pleasure, therefore, appears to be the result of one or more interrelated factors: eating being transformed into an imposed obligation; patients experiencing fear of eating; the senses involved in the eating process being ignored or overridden—with cold eating environments, devoid of sensory stimulation, and a lack of attention to taste and aesthetics in food presentation—due to new cooking techniques or the introduction of artificial feeding; and, finally, the social isolation experienced as a result of their new feeding needs.

## Figures and Tables

**Table 1:** Participants’ data and types of disability/illness.

	Count (*n*)
Gender	
Female	6
Male	21
Age	
Under 50	7
50 and over	20
Disability	
Brain injury	6
Spinal cord injury	5
Cancer	10
Amyotrophic lateral sclerosis	1
Multiple sclerosis	2
Muscular dystrophy	2
Niemann–Pick	1

**Table 2:** In-depth interviews: categories and guide questions.

Category	Guide Questions
Before vs. after diagnosis	Did you enjoy eating before your illness? What about now? Do you still eat the same kind of foods as before your illness was diagnosed? What types of foods have you stopped eating, and why?
Favourite foods	What are your favourite foods?
Attributes and importance of food	What do you think are the most important attributes of food? Are these attributes fulfilled by the foods you can consume now? What do you feel when a certain food fails to fulfil these attributes? Do you still need to consume those foods?
Visual aesthetics	Do you choose foods according to the way they look? Did you do so before your illness was diagnosed?
Significance	What does food mean to you? What do you feel when you see other people eating foods that you no longer can consume, because of your illness? Do you ever eat foods that you crave, despite knowing they might be harmful to you or affect the course of your illness?
Social environment and activities	Did you previously eat out often? Has anything changed since your illness was diagnosed?
Clinical decisions	Have you been recommended a gastrostomy? Have you accepted/rejected it? Why? What do you feel about meal replacement shakes? What about artificial feeding? What do you feel when the tube is introduced? How has it affected your life?

## Data Availability

The data presented in this study are available on request from the corresponding author. The data are not publicly available due to confidentiality agreements with participants.
